# Type 2 Diabetes in Relation to Hip Bone Density, Area, and Bone Turnover in Swedish Men and Women: A Cross-Sectional Study

**DOI:** 10.1007/s00223-018-0446-9

**Published:** 2018-06-26

**Authors:** Adam Mitchell, Tove Fall, Håkan Melhus, Alicja Wolk, Karl Michaëlsson, Liisa Byberg

**Affiliations:** 1 Department of Surgical Sciences, Orthopaedics, Uppsala University, Epihubben, Uppsala Science Park, 751 85 Uppsala, Sweden; 2 Department of Medical Sciences, Molecular Epidemiology, Uppsala University, Uppsala, Sweden; 3 Department of Medical Sciences, Clinical Pharmacogenomics and Osteoporosis, Uppsala University, Uppsala, Sweden; 4 Institute of Environmental Medicine, Division of Nutritional Epidemiology, Karolinska Institutet, Stockholm, Sweden

**Keywords:** Type 2 diabetes mellitus, Insulin, Glucose, Bone mineral area, Bone turnover markers

## Abstract

Men and women with type 2 diabetes mellitus (T2DM) have higher risk of hip fracture, but the mechanisms are not fully understood. We aimed to investigate how T2DM, glucose, and insulin were associated with femoral bone mineral density (BMD), bone mineral area (BMA), and bone turnover markers. We used two cross-sectional cohorts: the Uppsala Longitudinal Study of Adult Men (ULSAM, *n* = 452, mean age 82 years) and the Swedish Mammography Cohort Clinical (SMCC, *n* = 4713, mean age 68 years). We identified men and women with normal fasting glucose (NFG), impaired fasting plasma glucose (IFG), and T2DM. BMD and BMA at the total hip and femoral shaft were measured using dual energy X-ray absorptiometry (DXA). Bone turnover markers; CrossLaps and osteocalcin were measured in women. Linear regression models were applied. Men and women showed a progressively higher BMD following the clinical cutoffs of fasting glucose from NFG to IFG to T2DM. In contrast, there was a progressively lower BMA. Men and women with T2DM, compared to those with NFG, had lower BMA at the total hip (− 1.7%; 95% CI − 3.2, − 0.2 and − 1.0%; 95% CI − 1.6, − 0.4) and the femoral shaft (− 2.0%; 95% CI − 3.5, − 0.4 and − 0.6%; 95% CI − 1.2, − 0.01), respectively. T2DM was associated with lower concentrations of CrossLaps (− 8.1%; 95% CI − 12.7, − 3.6) and osteocalcin (− 15.2%; 95% CI − 19.0, − 11.2). These cross-sectional results indicate that those with T2DM have smaller bone area and lower bone turnover, which could increase the risk of hip fracture.

**Electronic supplementary material:**

The online version of this article (10.1007/s00223-018-0446-9) contains supplementary material, which is available to authorized users.

## Introduction

Individuals with type 2 diabetes mellitus (T2DM) are at an increased risk of fracture particularly at the hip, with similar risk in men and women [1]. Hip fractures present a great concern in the elderly population. They are associated with high healthcare costs [2] and an increase in the risk of death in the first year post fracture. The initial higher mortality is consistent in men and women independent of genes, comorbidity, and lifestyle [3].

A fracture is usually the consequence of a fall and the risk of fracture following a fall increases with advancing age and diminishing bone mineral density (BMD) [4]. The increased risk of fracture in those with T2DM persists despite a normal or higher BMD [5]. The pathophysiological mechanisms for this paradox remain unknown, but they can potentially be divided into mechanisms that weaken bone structure, and those that increase the likelihood of impaired balance and falls [6]. Bone size is an independent determinant of bone strength, and contributes to hip fracture risk independent of bone density [7]. Smaller bone width has been shown to be associated with increased risk of hip fracture in women and men [8, 9]. Recent evidence suggests that older adults with T2DM have a smaller bone area [10]; however, this has only been assessed at peripheral appendicular skeletal sites and not at the hip.

Further, there is a lack of understanding as to what happens in the transition from pre-diabetes to overt T2DM with regard to bone and fracture. Those with the metabolic syndrome commonly have a high BMI and insulin resistance and have a reduced risk of fracture [11]. Hyperglycemia may lead to the accumulation of advanced glycosylation end-products (AGEs) in the organic bone matrix, which could lead to biomechanically more brittle bone that is less able to deform before fracturing [12]. While insulin may have an anabolic effect on bone resulting in higher BMD [13], insulin resistance has been associated with smaller bone size [14, 15] at the tibia and distal radius. Data on the associations between glucose and insulin metabolism and bone area at the hip are lacking.

Throughout life, the skeleton undergoes continuous turnover of bone which allows the skeletal system to respond to outside mechanical forces or molecular signals [16] with a net loss of bone partially compensated by an increase in bone size with growing age [17]. Serum levels of the bone resorption marker C-terminal cross-linked telopeptide (CrossLaps) and the bone formation marker osteocalcin are lower in patients with diabetes [18], suggesting that T2DM may be a state of low bone turnover, potentially leading to a more brittle and fragile bone including also a theoretically negative effect on bone size by reduced periosteal bone formation with increasing age [17].

Using two Swedish population based cohorts, one in men and one in women, our main aim was to investigate the association between T2DM and femoral BMD and BMA measured by dual energy X-ray absorptiometry (DXA) at the hip. To help explain potential associations, we further examined whether T2DM, fasting glucose, and insulin were associated with biomarkers of bone turnover (among women).

## Materials and Methods

### Study Population

We included participants from two population-based cohorts based in central Sweden, Uppsala Longitudinal Study of Adult men (ULSAM) and Swedish Mammography Cohort Clinical (SMCC).

#### Men (ULSAM)

ULSAM was initiated in 1970 when all men born between 1920 and 1924, living in the county of Uppsala, Sweden, were invited to a health survey [19]. Of the 2322 men participating in the first examination cycle 34 reported diabetes and these were excluded to limit the potential for inclusion of those with type 1 diabetes. The men were regularly re-examined, and the current analyses were based on the 5th examination cycle in 2003–2005, when 952 of the original sample who were alive and still living in Uppsala county were invited for examination, and of these, 526 men (mean age 82 years) were examined. The examination was performed after an overnight fast and included blood sampling, measurement of height and weight, DXA measurements, and a medical and lifestyle questionnaire. Of these men, 455 had complete information on exposures and outcomes and had not reported diabetes at the first examination cycle. We excluded 3 men with missing information on covariates, which left 452 men (Online Resource 2). Further restriction to those without known T2DM or diabetes medication left 414 men for analysis of fasting glucose and insulin.

#### Women (SMCC)

The Swedish mammography cohort (SMC) was established during 1987–1990. All 90 303 women residing in two Swedish counties (Uppsala and Västmanland) born between 1914 and 1948 received a mailed invitation to a routine mammography screening. Between November 2003 and October 2009 a randomly selected subcohort (SMCC) of 5037 women living in the city of Uppsala underwent DXA measurements, provided morning fasting blood samples, had height and weight measurements taken, and completed a medical and lifestyle questionnaire [20]. Of these, 4713 women had given complete information on exposure and outcomes (Online Resource 3). Further restriction to women without known T2DM or diabetes medication left 4438 women with fasting glucose and 3917 with fasting insulin measurements.

### Exposures

#### Type 2 Diabetes Mellitus (T2DM), Impaired Fasting Glucose (IFG) and Normal Fasting Glucose (NFG)

We defined T2DM according to the World Health Organisation (WHO) [21] and American Diabetes Association (ADA) criteria using fasting plasma glucose ≥ 7.0 mmol/l and/or self-reported diabetes with or without treatment with oral hypoglycemic agents or insulin. Impaired fasting glucose (IFG) was defined according to the ADA criteria as fasting plasma glucose ≥ 5.6 mmol/l and normal fasting glucose (NFG) was defined as fasting plasma glucose as < 5.6 mmol/l.

#### Fasting Glucose and Insulin

##### Men (ULSAM)

In men, fasting plasma glucose concentrations (mmol/l) were measured by the glucose dehydrogenase method (Gluc-DH, Merck, Darmstadt, Germany). Fasting plasma insulin (mU/l) was assayed using an enzymatic-immunological assay (Mercodia Insulin ELISA, Uppsala, Sweden).

##### Women (SMCC)

Fasting plasma glucose and fasting serum insulin in women were analyzed using routine methods at the Department of Clinical Chemistry and Pharmacology (Uppsala University Hospital). Fasting plasma glucose was measured using three different methods depending on date of collection; glucose dehydrogenase reagent (Bergman & Beving, instrument Advia 1650, Stockholm, Sweden), glucose oxidase method (Bayer, instrument Advia 1650, Leverkusen, Germany), and hexokinase method (Abbott, Abbott Architect, Illinois, USA). For fasting serum insulin, again three different methods of enzymatic-immunological assay were used depending on date of collection: ADVIA Centaur Insulin (Bayer, Lerverkusen, Germany), Modular Insulin E170 (Roche Diagnostics, Rotkreuz, Switzerland), and Cobas8000 e620 (Cobas Elecsys Insulin reagent kit, Roche Diagnostics, Rotkreuz, Switzerland). In SMCC, certain fasting serum insulin samples were analyzed fresh, whereas others were stored, frozen − 80 °C, and then analyzed later. This resulted in slightly different mean values between the two protocols; therefore, in our statistical models, we adjusted for the differences in the analysis methods.

### Outcomes

#### Bone Mineral Density, Bone Mineral Area and Femoral Neck Diameter

**Fig. 1:**
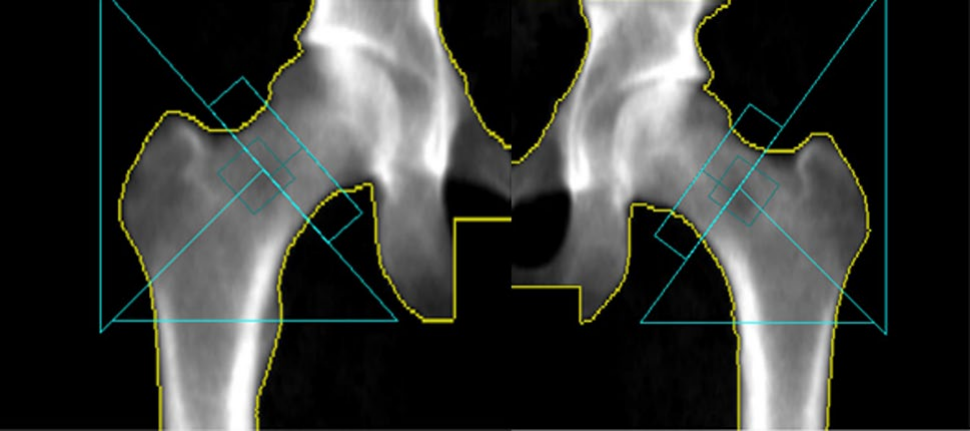
DXA image of dual femur and total hip in an adult male. Total hip area region of interest (ROI) is defined as the total area within the blue lines. Femoral shaft area ROI is defined as the total area within the blue lines of the bottom triangle. Femoral neck diameter ROI is defined as the diameter across the smallest part of the top rectangle. This is the standard output from the Lunar Prodigy DXA scanner

We measured bone mineral density (BMD, g/cm^2^) and bone mineral area (BMA, cm^2^) of the total hip and femoral shaft, by dual energy X-ray absorptiometry (DXA, DPX Prodigy, Lunar corp., Madison, WI, USA). These two sites were selected since there is a particularly strong association between T2DM and hip fracture risk [22], and as the total hip is constructed of both cancellous and cortical bone while the femoral shaft is dominated by cortical bone. We additionally measured the femoral neck diameter (mm). All measurements in both study populations were taken by the same experienced and accredited DXA X-ray nurse and using the same DXA machine. For the scan of the hip, the hip is in a standard position by a fixed position of the foot, ankle, and knee, to assure that area does not vary due to differences in rotation. That the rotation of the hip is accurate is always checked before the scan is accepted. The standard output from the DXA scanner provides the femoral neck, Ward’s area, trochanter, and femoral shaft region of interest (ROI), as shown in (Fig. 1). The femoral neck ROI is fixed at a 1.5 cm width and is positioned at the location where the product of bone mineral content (BMC) and area is lowest along the femoral neck and rotated to be perpendicular to the neck bone edges. The femoral neck diameter is the diameter across the femoral neck ROI. The Ward’s area is defined as a square positioned with 1/3 below the neck axis and 2/3 above the location of minimum BMC along the femoral neck; the square width is 0.5 times the average femoral neck width. The trochanter ROI is a triangular region with its most medial point placed at 1/6 of the Ward ROI size above the neck axis at the distal edge of the femoral neck ROI, extending from this point upwards along the distal edge of the neck ROI and downward at a 45° angle to the scan field. The femoral shaft area ROI is defined by the bottom of the trochanter ROI and the distal edge of the neck ROI with the shaft ROI base extending 5 cm down from the intersection of the trochanter ROI. The total hip area ROI is defined as the total area within the blue lines. If necessary, the region of interest (ROI) was adjusted to be the same location for each subject (approximately < 0.5% of scans). By triple measurements in 15 subjects, the precision error of the DXA measurements in our laboratory is 0.8–1.5% for BMD and 0.7–1.6% for bone areas depending on site.

#### Bone Turnover Markers

In women, fasting serum concentrations of CrossLaps and osteocalcin were analyzed on a Roche Cobas 8000 e602 module (Roche Diagnostics, Mannheim, Germany) using the β-CrossLaps and N-MID Osteocalcin reagent kits. The total coefficient of variation (CV) for CrossLaps was 1.8% at 294 ng/L and 1.4% at 2869 ng/L. CV for osteocalcin was 1% at 184 µg/L and 1% at 201 µg/L. Bone turnover markers were unavailable in men.

### Statistical Analysis

The baseline characteristics are presented as means (SD) and frequencies (%). All analyses were performed with linear regression conducted in crude (age-adjusted) models and multivariable adjusted models. Covariates included the continuous variables (measured at the clinical examination) age, height and BMI and the categorical variables (obtained from questionnaires) smoking status (never smoker, former smoker, current smoker), physical activity (1 lowest to 4 highest) and education (≤ 7, 8–10 and ≥ 12 years). In women, there were originally five categories of physical activity, but the two highest categories were collapsed. Covariates were selected based on the directed acyclic graph (DAG) method (Online Resource 5).

We analyzed the associations between NFG, IFG and T2DM, with BMD and BMA at the proximal femur and femoral shaft together with the femoral neck diameter. To quantify potential differences in BMD and BMA, we calculated a percentage difference by dividing the β estimate generated from the regression models by the mean value of either BMD or BMA at either the total hip or femoral shaft multiplied by 100. We further examined the association of fasting glucose and insulin as continuous variables with BMD and BMA, excluding those with prior T2DM diagnoses or diabetes treatment to ensure that diabetes treatment did not influence glucose, insulin, or the outcome measures. We repeated these analyses in women using the bone turnover markers CrossLaps and osteocalcin as outcomes.

For fasting glucose and insulin, we first analyzed the linear associations and then, to further examine potential nonlinear associations, we used restricted cubic splines using three “knots” placed at centiles 10, 50, and 90 of fasting glucose and insulin. These centiles corresponded to 4.8, 5.6, and 6.8 mmol/L for fasting plasma glucose and 3.1, 6.0, and 12.3 mU/l for fasting plasma insulin in men; and 4.49, 5.12, and 5.9 mmol/L for fasting plasma glucose and 3.1, 6.09, and 12.67 mU/l for fasting serum insulin women. For fasting serum insulin in women, we included an indicator variable for the method of insulin analysis as a covariate.

In sensitivity analyses, we adjusted associations for fasting glucose by fasting insulin and vice versa, and height and weight were included as covariates instead of height and BMI. To exclude that area measurements were influenced by different levels of obesity, we examined associations between NFG, IFG, and T2DM with BMD and BMA among those women with BMI 25–29.9 kg/m^2^ (this analysis was not performed in men, since the sample size was too small to perform subgroup analysis). We performed a complete case analysis; therefore, those with missing data in any of the variables were excluded. Missing data ranged from 6 to 14%. In sensitivity analysis, we used multiple imputations for missing covariates which did not affect the estimates.

All statistical analyses were conducted using StataSE 14 software for Windows.

## Results

The characteristics of subjects in each cohort are presented in Table 1 and displayed by clinical categories of fasting plasma glucose status. The mean age of men was 82 years ranging between 80 and 84 years, and the mean age of women was 68 years ranging from 55 to 86 years. 15% of men and 7% of women had T2DM. Comparing those with T2DM to those with NFG, they had greater BMI, lower attained educational level, and were less physically active. The mean values for outcome variables are presented in Table 2.

**Table 1:** Population characteristics

	ULSAM (men)			SMCC (women)		
	NFG	IFG	T2DM	NFG	IFG	T2DM
*N* (%)	234 (51.8)	150 (33.2)	68 (15.0)	3591 (76.2)	797 (16.9)	325 (6.9)
Age, mean (SD)	81.5 (0.9)	81.8 (1.0)	81.8 (1.0)	67.1 (6.5)	67.6 (6.9)	69.2 (7.2)
Height (mean, cm)	172.8 (5.4)	172.8 (6.0)	173.5 (4.8)	163.8 (6.1)	163.6 (6.0)	162.7 (6.0)
BMI, kg/m^2^	25.4 (3.1)	26.3 (3.4)	27.1 (3.8)	25.3 (3.9)	27.4 (4.7)	28.9 (5.5)
Smoking status, *n* (%)						
Never smoker	105 (44.9)	65 (43.3)	24 (35.3)	2060 (57.4)	410 (51.4)	188 (57.9)
Former smoker	112 (47.9)	78 (52.0)	41 (60.3)	1204 (33.5)	310 (38.9)	113 (34.8)
Current smoker	17 (7.2)	7 (4.7)	3 (4.4)	327 (9.1)	77 (9.7)	24 (7.3)
Physical activity, *n* (%)						
1 (lowest)	29 (12.4)	29 (19.3)	12 (17.7)	573 (15.9)	132 (16.6)	70 (21.5)
2	85 (36.3)	54 (36.0)	24 (35.3)	756 (21.1)	197 (24.7)	78 (24.0)
3	106 (45.3)	62 (41.3)	30 (44.1)	1252 (34.9)	278 (34.9)	95 (29.2)
4 (highest)	14 (6.0)	5 (3.3)	2 (2.9)	1010 (28.1)	190 (23.8)	82 (25.2)
Education, *n* (%)						
>7 years	126 (53.8)	84 (56.0)	43 (63.2)	1838 (51.2)	456 (57.2)	212 (65.2)
8–10 years	46 (19.7)	33 (22.0)	11 (16.2)	340 (9.5)	55 (6.9)	20 (6.2)
<12 years	62 (26.5)	33 (22.0)	14 (20.6)	1413 (39.3)	286 (35.9)	93 (28.6)
Fasting glucose (mean, mmol/l)	5.2 (0.4)	6.2 (0.4)	8.4 (1.4)	5.0 (0.4)	6.0 (0.3)	7.6 (2.5)
Fasting insulin (mean, mU/l)	6.3 (3.5)	8.3 (5.8)	10.6 (7.8)	6.6 (4.0)^a^	10.6 (6.8)^a^	14.9 (17.0)^a^

^a^SMCC. Fasting serum insulin. NFG *n* = 3197. IFG *n* = 679. T2DM *n* = 273

**Table 2:** Population outcome averages

Total hip	Men (*n* = 452)		Women (*n* = 4713)	
	Mean	SD	Mean	SD
BMD (g/cm^2^)	0.99	0.16	0.92	0.13
BMA (cm^2^)	39.3	2.71	32.5	2.20
Femoral shaft				
BMD (g/cm^2^)	1.14	0.19	1.08	0.17
BMA (cm^2^)	15.9	1.08	14.5	0.85
Bone TM				
Crosslaps (ng/ml)			460.7^a^	191.7
Osteocalcin (µg/l)			25.0^b^	8.89

*TM* turnover marker

^a^SMCC. CrossLaps *n* = 5000

^b^Osteocalcin *n* = 4998

**Fig. 2:**
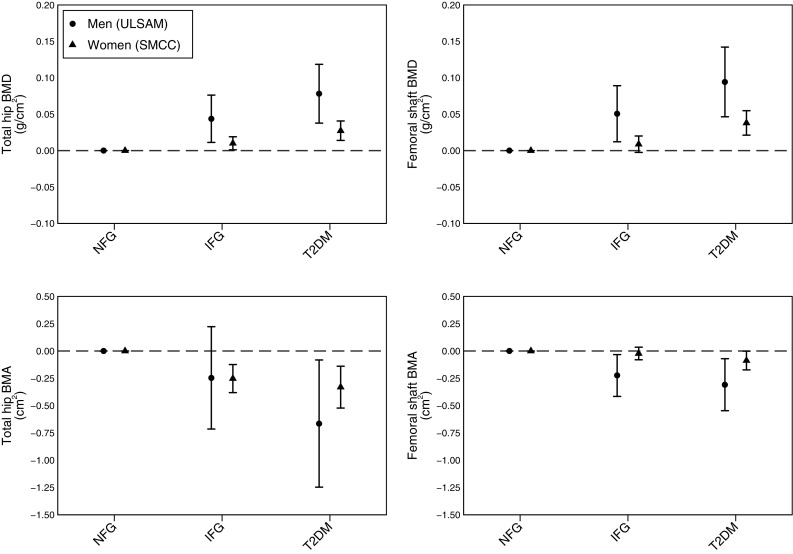
Differences in BMD and BMA at the total hip and femoral shaft between clinical categories of fasting glucose. The differences in bone mineral density (BMD) and bone mineral area (BMA) measured at the total hip and femoral shaft between clinical categories of fasting plasma glucose; normal fasting glucose (NFG), impaired fasting glucose (IFG), type 2 diabetes mellitus (T2DM) in men (ULSAM; Uppsala Longitudinal Study of Adult Men) and women (SMCC; Swedish Mammography Cohort Clinical). Numbers presented are β estimates and 95% confidence intervals from linear regression analysis and adjusted for age, height, body mass index, smoking status, physical activity, and education

In fully adjusted models, there was a progressively higher BMD following the clinical cutoffs of fasting glucose from NFG to IFG to T2DM. In contrast, there was progressively a lower BMA following the clinical cutoffs of fasting glucose. As shown in (Fig. 2), T2DM was associated with greater BMD corresponding to an increase of 8.0% (95% CI [4.0, 12.0]) and 7.9% (95% CI [4.4, 12.3]) at the total hip and femoral shaft, respectively, in males; and 3.3% (95% CI [1.1, 4.4]) and 3.7% (95% CI [1.9, 5.6]) in females. However, conversely, T2DM was associated with lower BMA corresponding to − 1.7% (95% CI [− 3.2, − 0.2]) and − 2.0% (95% CI [− 3.5, − 0.4]) at the total hip and femoral shaft, respectively, in males; and − 1.0% (95% CI [− 1.6, − 0.4]) and − 0.6% (95% CI [− 1.2, − 0.01]) in females (Fig. 2). Those with IFG also presented with a lower BMA corresponding to − 0.6% (95% CI [− 1.8, 0.6]) and − 1.4% (95% CI [− 2.7, − 0.2]) at the total hip and femoral shaft, respectively, in males; and − 0.8% (95% CI [− 1.2, − 0.4]) and − 0.1% (95% CI [− 0.6, 0.3]) in females (Fig. 2). The same direction of association was seen when we measured the association between the clinical cutoffs and the femoral neck diameter (mm) (Online Resource 6). We further restricted the analysis to women with BMI ≥ 25 to 29.9 kg/m^2^ and report the same direction of association between T2DM and BMA (Online Resource 7). This was also the case when we adjusted for height and weight individually instead of height and BMI (Online Resource 8). Both fasting glucose and fasting insulin were inversely associated with BMA at the total hip and femoral shaft (Online Resources 1 & 4).

**Fig. 3:**
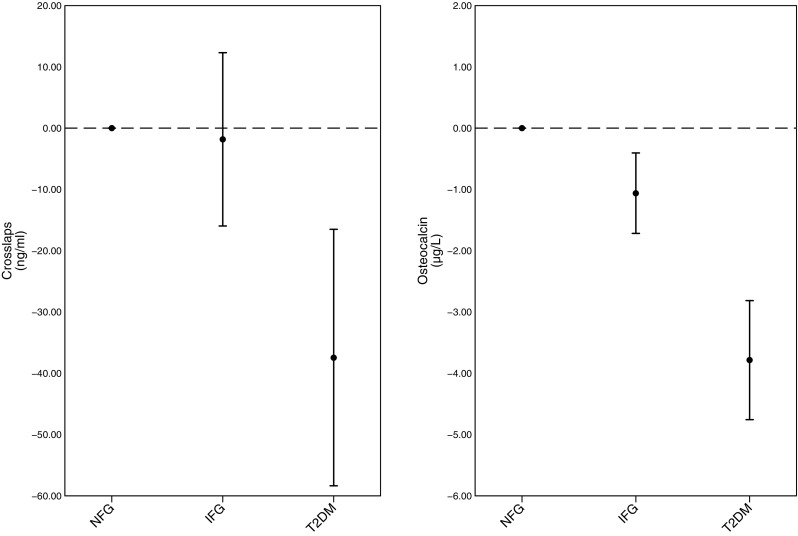
Differences in CrossLaps and Osteocalcin between clinical categories of fasting plasma glucose. The differences in bone turnover markers: CrossLaps and between clinical categories of fasting plasma glucose; normal fasting glucose (NFG), impaired fasting glucose (IFG), type 2 diabetes mellitus (T2DM) in women (SMCC; Swedish Mammography Cohort Clinical). Numbers presented are β estimates and 95% confidence intervals from linear regression analysis and adjusted for age, height, body mass index smoking status, physical activity, and education

**Fig. 4:**
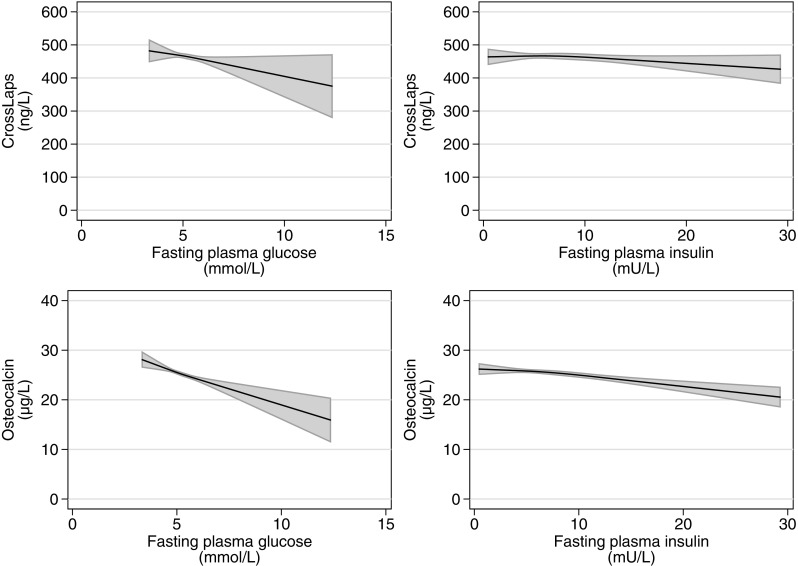
Association between fasting glucose, fasting insulin, and CrossLaps and Osteocalcin. Restricted cubic splines showing the association between fasting plasma glucose and fasting plasma insulin, and CrossLaps and Osteocalcin in women of the SMCC (Swedish Mammography Cohort Clinical). Models adjusted for age, height, body mass index, smoking status, physical activity, and education. Insulin models additionally adjusted for method of analysis in SMCC

In women, IFG was associated with a lower bone formation (osteocalcin) corresponding to − 4.2% (95% CI [− 6.9, − 1.6]), and T2DM was associated with lower concentrations of both CrossLaps and osteocalcin, corresponding to − 8.1% (95% CI [− 12.7, − 3.6]) and − 15.2% (95% CI [− 19.0, − 11.2]) (Fig. 3). Fasting glucose and insulin were also inversely associated with CrossLaps and osteocalcin in those without known T2DM (Fig. 4). A 1 mmol/L increase in fasting glucose was associated with lower concentrations of CrossLaps corresponding to − 2.5% (95% CI [− 4.3, − 0.6]) and lower concentrations of osteocalcin corresponding to − 5.3% (95% CI [− 6.8, − 3.7]). A 1 mU/l increase in fasting insulin was associated with lower concentrations of osteocalcin corresponding to − 0.60% (95% CI [− 0.8, − 0.4]) (Fig. 4).

Sensitivity analyses adjusted for fasting glucose for insulin and vice versa did not alter estimates (results not shown).

## Discussion

Our cross-sectional analyses based on one cohort of Swedish men and one of Swedish women show that in addition to the higher BMD also previously seen among those with T2DM, they also present with a smaller area at the total hip and the femoral shaft. We also observed that fasting glucose and insulin were associated with smaller BMA. Among women, T2DM, higher levels of both fasting glucose and insulin were also associated with lower levels of bone turnover markers.

There is substantial evidence that older adults with T2DM have a higher risk of hip fracture [22] consistent in both men (RR 2.8) and women (RR 2.1) [23]. Despite this increased risk of fracture, our results comply with previous research to show that patients with T2DM have normal or greater BMD than those without T2DM [5]. There are previous reports on T2DM being associated with smaller bone area; however, they all measured bone at peripheral sites using high resolution peripheral quantitative computed tomography (pQCT). The bone area at the proximal femur and femoral shaft are most relevant for hip fracture risk which is the most devastating fracture. T2DM was associated with a smaller cross-sectional area at the tibia and radius (men and women *n* = 1069) [10], smaller cortical area of the tibia (25 postmenopausal women with T2DM and 25 controls) [24] and lower endosteal and periosteal circumferences and total area at the tibia (hypogonadal males *n* = 105) [25]. In addition to measuring bone area at peripheral sites, these studies were conducted in relatively small and specific populations, potentially restricting the generalizability of the results.

Bone size has been shown to be an independent determinant of bone strength [26], and the biomechanical strains from enhanced physical activity may promote increases in bone size that in turn can help preserve bone strength [27]. In women, bone area and body size factors contribute to hip fracture risk independent of bone density [7], and a smaller femoral neck diameter is associated with the increased risk of hip fracture [8]. Further, males with hip fractures had smaller femoral neck width and vertebral body width [9], and from the structural analysis of the hip using pQCT, cross-sectional area of the femoral neck may be a predictor of hip fracture risk [28]. Biomechanically, there is a strong relation between bending strength and bone diameter, and small differences in area can lead to large differences in strength. Indices of femoral neck strength have been shown to be inversely associated with incident hip fracture risk in older Caucasian women [7], and bending strength at the femoral neck has been shown to be reduced in those with T2DM, with insulin resistance reported as an important factor in this association [29].

With advancing age, bone size tends to increase which can partially compensate for the loss in BMD. Bone area increases equally over life in both sexes by ~ 15% at central sites and by ~ 16% at peripheral sites [30]. Decreases in trabecular volumetric BMD (vBMD) begin before midlife and continue throughout life, whereas decreases in cortical vBMD begin in midlife [30]. We observed lower BMA at the proximal femur and femoral shaft with higher levels of fasting glucose. There are currently no studies showing an association between glucose and bone area; however, hyperglycemia has been shown to instigate a higher concentration of AGEs in collagen [12]. High levels of glucose have also been shown to reduce expression of the transcription factor RUNX2 and inhibit bone formation [31].

We also found that high levels of insulin, often used as a proxy for insulin resistance, were associated with lower BMA. Insulin resistance measured using HOMA-IR has been shown to be inversely associated with bone size [14], potentially explained by high levels of insulin being inversely associated with sex hormone-binding globulin (SHBG), thus increasing the free concentrations of androgens and estrogens [32]. However, insulin resistance was associated with smaller bone size independent of sex steroid levels in younger adults (mean age 34.5 years) [33].

T2DM has in meta-analyses been associated with lower levels of the bone turnover markers; osteocalcin, CrossLaps, and procollagen type 1 amino terminal propeptide [18, 34] indicating a lower bone turnover. We similarly observe lower levels of osteocalcin and CrossLaps among women with T2DM. Animal and cell culture studies suggest that hyperglycemia can affect bone tissue as well as bone turnover [35, 36] and serum levels of osteocalcin have also been shown to be inversely associated with fasting insulin and insulin resistance in humans [37]. Mechanisms to explain this observation come from in vitro studies, which report that hyperglycemia increases sclerostin expression by osteocyte cell lines [38]. Sclerostin is a monomeric glycoprotein expressed by the *SOST* gene in osteocytes and appears to play an important role in the regulation of bone remodeling by inhibiting canonical Wnt/β-catenin signaling, particularly in those with T2DM [39]. Postmenopausal women with T2DM had higher serum sclerostin accompanied by decreased PTH levels that resulted in low bone turnover [40]. It is suggested that low bone turnover in T2DM is associated with lower mineralizing surface of bone and lower osteoid surface quality [41], which may help further explain the higher risk of fracture in those with T2DM.

The main strength of this study is that fasting glucose, insulin, BMD and BMA were measured in two large population-based cohorts consisting of men and women, both with and without T2DM. In addition, markers of bone turnover were measured in women. We are unaware of other studies combining several bone outcomes, in particular BMA at the hip, in relation to T2DM, glucose, and insulin in this manner. Although the two cohorts included in our study were examined at different time points and one included men and one included women of different ages, the fact that we see similar results in these two different populations may be seen as a strength of the study findings and may increase their generalizability. The comparability of the populations and the interpretation of the DXA outcomes is, however, facilitated by the fact that all DXA measurements were taken by the same nurse using the same DXA scanner. Unfortunately, bone turnover markers were not available in the male cohort. The large number of participants with DXA measurements and with fasting samples of glucose and insulin is unique. The fasting samples enabled clinical categorization of individuals with T2DM according to concentrations of fasting glucose in combination with self-reported diagnosis and medication use. However, we only used one measure of fasting plasma glucose, which may have led to misclassification of T2DM.

The main limitation of our study is that bone mineral area (BMA) was measured using the two dimensional DXA technique set at a specific ROI. The technique restricts our ability to distinguish between cortical and trabecular bone and measure the microarchitecture and quality of bone mineralization [42], leading to potentially conservatively biased estimates. Three-dimensional imaging techniques [43] are the desirable method to overcome some of these limitations. However, the most commonly used pQCT is a peripheral measurement tool and DXA of the total hip may be a more accurate way to rank individuals according to bone mineral area at the hip. Body weight may influence the distance between the DXA bed and the bone measured; however, to minimize the impact of such differences, the Lunar Prodigy DXA used in the current study corrects the scans to the actual effective object plane. Differences in height between 5 and 15 cm results in an uncertainty of 1% for area measurements [44]. The influence of tissue thickness is also minimal [44, 45]. Furthermore, magnification effects are smaller using the narrow fan-beam along the axis as in the Lunar Prodigy, compared to wide-angle fan beam with perpendicular orientation. These beam-related features of the DXA scanner used, in addition to high resolution, automatic location of the bone, and centering of the scan around the bone providing precise automatic edge detection, gives an improved measurement less dependent of the exact positioning of the femur in the beam and no scout scans are needed [44, 45]. Thus, the problem with high body weight when determining bone size is of less importance with this equipment compared with ordinary fan-beam equipment [44, 45]. The ordinary fan-beam equipment can have substantial magnification error, which has direct effects on estimated bone area [44]. Body weight may also influence the individual’s position on the DXA scanner and therefore the area; however, we used a standard position for each subject that was checked before accepting each scan. Therefore we believe that the different positioning of the hip or differences in body weight alone would explain the differences in bone area by glycemic status is unlikely. In sensitivity analysis among women who were overweight, results were similar to those in the main results indicating that the differences in bone area persist in those with similar body stature. Interindividual variation was also limited since each subject was measured by the same experienced and DXA-accredited X-ray nurse using the same scanner [44] to ensure the ROI consistency. The nurse was unaware of the participants’ glycemic status at the time of measurement, and any differences in positioning, which would therefore introduce random measurement error, were unlikely to bias the results. Despite the limitations of using DXA as a measurement of BMA, our results are in the same direction as other studies using pQCT methods. In our study, there were 68 (15%) men and 325 (6.9%) women with T2DM, and the limited number of men in particular with T2DM may limit the power of our study. However, the percentage of individuals with T2DM is reflective of the prevalence of T2DM in Sweden at the respective ages [46]. The analyses using fasting concentrations of glucose as a continuous measure have higher power, and the results are in the same direction as the clinical categories of T2DM and IFG. Further limitations of our study include the cross-sectional design which limits our ability to infer causation.

## Conclusion

In conclusion, the results of our cross-sectional study suggest that elderly men and women with T2DM have greater BMD yet a lower BMA at the hip and femoral shaft as well as lower bone turnover. This lower BMA together with a lower bone turnover may help to provide more understanding as to why those with T2DM have an increased risk of hip fracture despite higher BMD.

## Electronic supplementary material

Below is the link to the electronic supplementary material.

Supplementary material 1 (DOCX 18 KB)

Supplementary material 2 (DOCX 23 KB)

Supplementary material 3 (DOCX 29 KB)

Supplementary material 4 (DOCX 43 KB)

Supplementary material 5 (DOCX 725 KB)

Supplementary material 6 (DOCX 15 KB)

Supplementary material 7 (DOCX 14 KB)

Supplementary material 8 (DOCX 17 KB)

## Abbreviations

ULSAM: Uppsala longitudinal study of adult men
SMCC: Swedish mammography cohort clinical
T2DM: Type 2 diabetes mellitus
IFG: Impaired fasting glucose
NFG: Normal fasting glucose
BMA: Bone mineral area
AGEs: Advanced glycosylation end-products
ADA: American Diabetes Association
