# Supplementary material for: Type 2 Diabetes in Relation to Hip Bone Density, Area, and Bone Turnover in Swedish Men and Women: A Cross-Sectional Study
Source: Calcif Tissue Int. 2018 Jun 26;103(5):501–11. doi: 10.1007/s00223-018-0446-9 (PMC6182615; doi:10.1007/s00223-018-0446-9)
Supplement: Supplementary file 3 — Supplementary material 3 (DOCX 29 KB) [file 223_2018_446_MOESM3_ESM.docx]

**Type 2 diabetes in relation to hip bone density, size and bone turnover in elderly Swedish men and women**

**Calcified Tissue International**

**Adam Mitchell ^1^, Tove Fall ^2^, Håkan Melhus ^3^, Alicja Wolk ^1,4^, Karl Michaëlsson ^1^, Liisa Byberg ^1^**

**Institutions of origin:**

1. Department of Surgical Sciences, Orthopaedics, Uppsala University, Sweden

2. Department of Medical Sciences, Molecular Epidemiology, Uppsala University, Sweden

3. Department of Medical Sciences, Clinical Pharmacogenomics and Osteoporosis, Uppsala University, Sweden

4. Institute of Environmental Medicine, Division of Nutritional Epidemiology, Karolinska Institutet, Sweden

**Corresponding author:**

Adam Mitchell

UCR/MTC, Uppsala Science Park

751 85 Uppsala, Sweden

[Adam.mitchell@surgsci.uu.se](mailto:Adam.mitchell@surgsci.uu.se)

Phone: +46 762561548

n=5022

Missing blood samples (n=15)

DXA missing (n=273)

(n=4713)

(n=4438)

T2DM not already diagnosed or on medication (n=275)

Fasting insulin missing (n=521)

(n=3917)

Crosslaps (n=5000)

Osteocalcin (n=4998)

Missing CrossLaps (n=22)

(n=4710)

(n=4708)

(n=4150)

(n=4148)

T2DM not already diagnosed or on medication (n=290)

Fasting insulin missing (n=560)

SMCC baseline (n=5037)

Missing Osteocalcin (n=24)

n=4986

Fasting glucose missing (n=36)

**Online Resource 3** SMCC flow chart of subject numbers for analysis
