# Supplementary material for: Type 2 Diabetes in Relation to Hip Bone Density, Area, and Bone Turnover in Swedish Men and Women: A Cross-Sectional Study
Source: Calcif Tissue Int. 2018 Jun 26;103(5):501–11. doi: 10.1007/s00223-018-0446-9 (PMC6182615; doi:10.1007/s00223-018-0446-9)
Supplement: Supplementary file 5 — Supplementary material 5 (DOCX 725 KB) [file 223_2018_446_MOESM5_ESM.docx]

**Type 2 diabetes in relation to hip bone density, size and bone turnover in elderly Swedish men and women; a cross-sectional study**

**Calcified Tissue International**

**Adam Mitchell ^1^, Tove Fall ^2^, Håkan Melhus ^3^, Alicja Wolk ^1,4^, Karl Michaëlsson ^1^, Liisa Byberg ^1^**

**Institutions of origin:**

1. Department of Surgical Sciences, Orthopaedics, Uppsala University, Sweden

2. Department of Medical Sciences, Molecular Epidemiology, Uppsala University, Sweden

3. Department of Medical Sciences, Clinical Pharmacogenomics and Osteoporosis, Uppsala University, Sweden

4. Institute of Environmental Medicine, Division of Nutritional Epidemiology, Karolinska Institutet, Sweden

**Corresponding author:**

Adam Mitchell

UCR/MTC, Uppsala Science Park

751 85 Uppsala, Sweden

[Adam.mitchell@surgsci.uu.se](mailto:Adam.mitchell@surgsci.uu.se)

Phone: +46 762561548


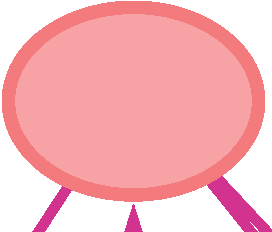


Physical Activity


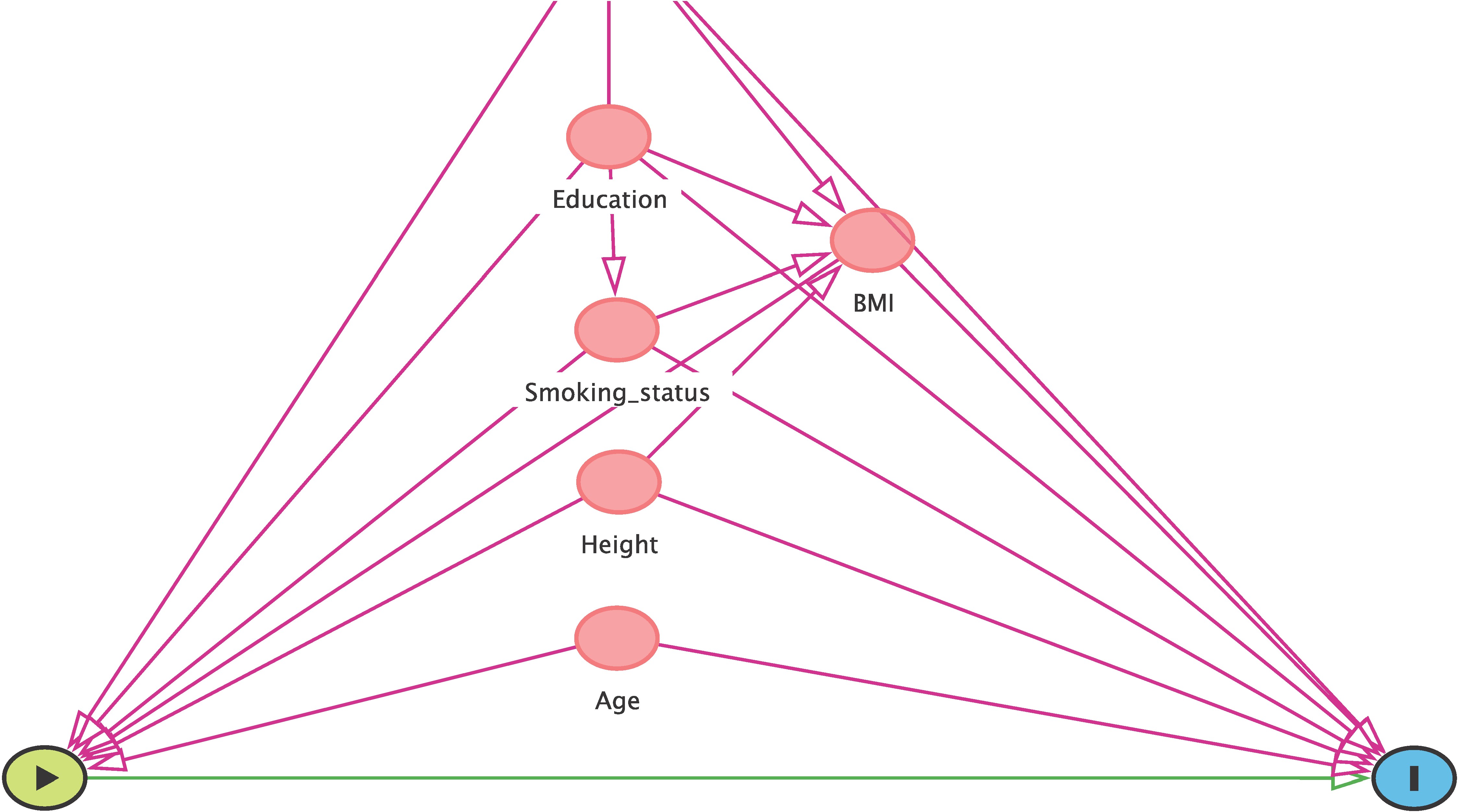


T2DM **BMA**

**Online resource 5** Directed acyclic graph (DAG) to determine the covariates used in the analysis.
