# Supplementary material for: Type 2 Diabetes in Relation to Hip Bone Density, Area, and Bone Turnover in Swedish Men and Women: A Cross-Sectional Study
Source: Calcif Tissue Int. 2018 Jun 26;103(5):501–11. doi: 10.1007/s00223-018-0446-9 (PMC6182615; doi:10.1007/s00223-018-0446-9)
Supplement: Supplementary file 7 — Supplementary material 7 (DOCX 14 KB) [file 223_2018_446_MOESM7_ESM.docx]

**Type 2 diabetes in relation to hip bone density, size and bone turnover in elderly Swedish men and women; a cross-sectional study**

**Calcified Tissue International**

**Adam Mitchell ^1^, Tove Fall ^2^, Håkan Melhus ^3^, Alicja Wolk ^1,4^, Karl Michaëlsson ^1^, Liisa Byberg ^1^**

**Institutions of origin:**

1. Department of Surgical Sciences, Orthopaedics, Uppsala University, Sweden

2. Department of Medical Sciences, Molecular Epidemiology, Uppsala University, Sweden

3. Department of Medical Sciences, Clinical Pharmacogenomics and Osteoporosis, Uppsala University, Sweden

4. Institute of Environmental Medicine, Division of Nutritional Epidemiology, Karolinska Institutet, Sweden

**Corresponding author:**

Adam Mitchell

UCR/MTC, Uppsala Science Park

751 85 Uppsala, Sweden

[Adam.mitchell@surgsci.uu.se](mailto:Adam.mitchell@surgsci.uu.se)

Phone: +46 7625615

**Online resource 7** Association between normal fasting glucose (NFG), impaired fasting glucose (IFG) and type 2 diabetes mellitus (T2DM) and bone mineral density (BMD), bone mineral area (BMA) in body mass index (BMI) category >25.0 – 29.9 (kg/m2)

|  | SMCC | |
| --- | --- | --- |
| **Total hip BMD** | Age | Full |
| N | 1800 | 1800 |
| NFG (ref) |  |  |
| IFG | 0.02 [0.00,  0.03] | 0.01 [0.00,  0.03] |
| T2DM  **Femoral Shaft BMD**  N  NFG (ref)  IFG  T2DM | 0.04 [0.02,  0.06]  1800  0.02 [0.00,  0.04]  0.05 [0.20,  0.07] | 0.03 [0.01,  0.06]  1800  0.02 [-0.00  0.03]  0.04 [0.01,  0.07] |
| **Total Hip BMA** |  |  |
| N | 1800 | 1800 |
| NFG (ref) |  |  |
| IFG | -0.03 [-0.30,  0.22] | -0.28 [-0.47,  -0.09] |
| T2DM | -0.20 [-0.57,  0.21] | -0.39 [-0.70,  -0.09] |
| **Femoral shaft BMA** |  |  |
| N | 1800 | 1800 |
| NFG (ref) |  |  |
| IFG | 0.02 [-0.10,  0.12] | -0.05 [-0.14,  0.03] |
| T2DM | -0.01 [-0.20,  0.15] | -0-05 [-0.20,  0.08] |

Age adjust. Adjusted for age only. Full. Adjusted for age, height, BMI, smoking status, physical activity and education.
